# Circadian and sleep–wake rhythm alterations in isolated REM sleep behavior disorder: biomarkers of prodromal α-synucleinopathy

**DOI:** 10.3389/fnins.2025.1727683

**Published:** 2025-12-16

**Authors:** Matteo Carpi, Claudio Liguori

**Affiliations:** 1 Department of Human Neuroscience, Sapienza University of Rome, Rome, Italy; 2 Neurology Unit, University Hospital of Rome Tor Vergata, Rome, Italy; 3 Department of Systems Medicine, University of Rome Tor Vergata, Rome, Italy

**Keywords:** biomarkers, circadian rhythms, isolated REM sleep behavior disorder (iRBD), melatonin, rest–activity rhythms, α-synucleinopathies

## Abstract

Growing evidence highlights a tight interplay linking circadian and sleep–wake disturbances to the pathophysiology of neurodegenerative disorders. In α-synucleinopathies, three key points have emerged: (1) circadian and sleep–wake disruptions may increase the risk of neurodegeneration; (2) these alterations reflect widespread dysfunction in neural circuits regulating sleep, wakefulness, and biological rhythms; and (3) the prodromal condition of isolated rapid eye movement (REM) sleep behavior disorder (iRBD) offers a unique window into early pathological changes, as it is characterized by neurodegeneration in brainstem structures critical for sleep–wake regulation and REM sleep control. Hence, sleep- and circadian-related biomarkers may represent feasible tools for early diagnosis, prevention, and treatment across the spectrum of α-synucleinopathies. However, despite their potential, diagnostic or therapeutic pathways grounded in sleep and circadian biology have yet to be systematically explored or validated, and key questions remain, including the trajectories that characterize the clinical progression from iRBD to overt α-synucleinopathies. Key challenges include translational barriers, inter-individual variability in biomarker profiles, and the need for longitudinal studies to define clinically actionable thresholds. Against this backdrop, this mini-review synthesizes current evidence on sleep–wake rhythm alterations in iRBD as a prodromal stage of α-synucleinopathy-driven neurodegeneration. Candidate circadian biomarkers are discussed, including objective parameters from long-term actigraphic monitoring, encompassing rest–activity rhythms modeled with parametric and non-parametric approaches, as well as physiological indicators such as dim light melatonin onset and core body temperature.

## 1 Introduction

**Figure 1:**
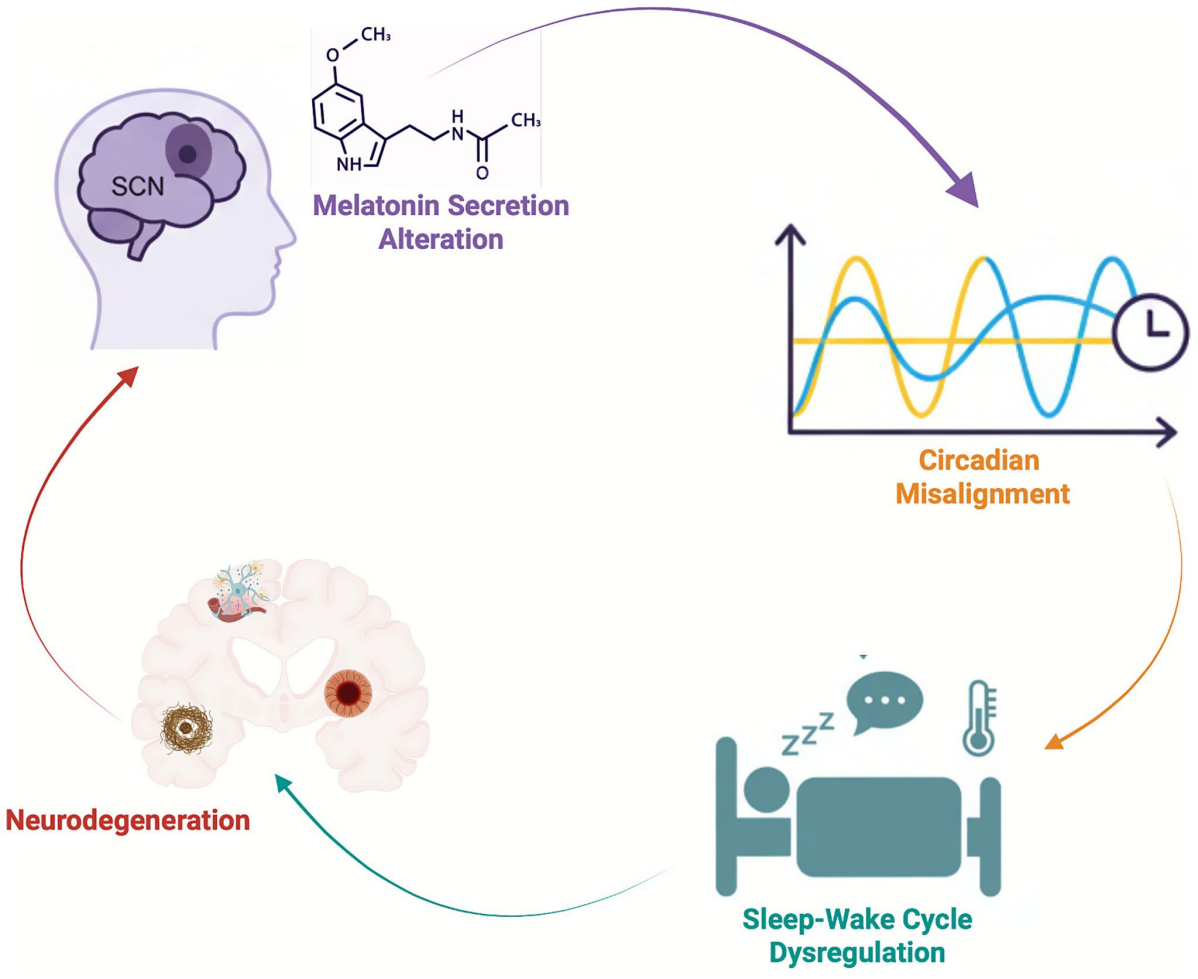
Conceptual model of potential links between circadian disruption and neurodegeneration in α-synucleinopathies. The suprachiasmatic nucleus (SCN) regulates melatonin secretion and synchronizes daily rhythms of physiology and behavior. Disruption in SCN signaling or its downstream pathways leads to circadian misalignment, marked by instability or phase shifts in endogenous rhythms, which may contribute to impaired sleep–wake regulation with downstream effects on arousal systems, thermoregulation, and REM sleep circuitry. These alterations, in turn, may promote (or reflect) early neurodegenerative processes in isolated rapid eye movement (REM) sleep behavior disorder (iRBD) and related α-synucleinopathies. Neurodegeneration may further impair SCN function and circadian regulation, creating a recursive feedback loop.

Alpha-synucleinopathies, including those conditions broadly characterized by abnormal pathological accumulation of α-synuclein protein in the brain—such as Parkinson’s disease (PD), dementia with Lewy bodies (DLB), and multiple system atrophy (MSA, which in contrast is marked by abnormal α-synuclein in the form of oligodendroglial cytoplasmic inclusions rather than neuronal Lewy bodies; Reddy and Dieriks, 2022)—are disabling diseases with rising prevalence and public health impact (Savica et al., 2013; Dorsey and Bloem, 2018). This increase, translating into high rates of disability and dementia (GBD 2019 Dementia Forecasting Collaborators, 2022) has fueled major interest in identifying biomarkers that can predict early disease trajectories and provide feasible targets for disease-modifying interventions (Savica et al., 2018). Within this context, sleep–wake and circadian disturbances are increasingly recognized as core features of α-synucleinopathies, representing burdensome and often early manifestations of these disorders rather than secondary phenomena (Chahine et al., 2017; Stefani and Högl, 2020; Elder et al., 2022; Kunz et al., 2023; Pounders and McCarter, 2025). From early reports to recent reviews, evidence has converged on a bidirectional relationship with pathology: neurodegeneration disrupts circadian and sleep regulation, while altered rhythms in turn aggravate disease mechanisms and symptom burden (Videnovic et al., 2014a; Minakawa, 2022; Nassan and Videnovic, 2022; Kunz et al., 2023) (see Figure 1 for a conceptual overview of these mechanisms).

The human circadian timing system is governed by a central “master clock” located in the suprachiasmatic nucleus (SCN) of the anterior hypothalamus, which synchronizes around 24-h cycles of physiology and behavior (Golombek and Rosenstein, 2010; Nassan and Videnovic, 2022). Through self-sustained molecular oscillations, the SCN drives daily fluctuations in hormone secretion, metabolism, and neuronal excitability, ultimately regulating outputs such as the sleep–wake cycle, melatonin secretion, and core body temperature. For example, melatonin, a hormone produced by the pineal gland, exhibits a robust and measurable nocturnal rise that serves as a well-established marker of internal circadian phase, and core body temperature also follows a predictable diurnal pattern, peaking in the late afternoon and reaching a nadir during the night under SCN influence. Notably, these rhythmic processes can be externally assessed through biological sampling or long-term actigraphy.

In this context, a sleep condition characterized by loss of physiologic muscle atonia during rapid eye movement (REM) sleep, leading to enactment of dream content, isolated REM sleep behavior disorder (iRBD), has been widely identified as a prodromal manifestation of α-synuclein-related pathology (Savica et al., 2018; Boeve and Ferman, 2024), and provides a valuable model for understanding the early neurodegenerative processes leading to full-blown PD, DLB, or MSA (Boeve et al., 2013; Postuma et al., 2019). Historically, the initial link between iRBD and Parkinson’s disease was proposed in a case series in the late 1990s (Schenck et al., 1996) and later confirmed in a 16-year longitudinal follow-up spanning approximately 30 years (Schenck et al., 2013), with converging evidence further substantiating this prodromal relationship since then. While post-mortem investigations have shown α-synuclein-related neuropathology in some iRBD patients who did not develop an overt neurodegenerative condition (Mayà et al., 2024), longitudinal evidence consistently indicates high conversion rates from iRBD to α-synucleinopathies, with pooled risks approaching 80% by 10 years and increasing with longer follow-up (Iranzo et al., 2014; Savica et al., 2018; Galbiati et al., 2019). Preclinical phases lasting up to 25 years have also been described (Claassen et al., 2010), further underscoring the need for scalable markers that stratify risk and track trajectories before clinical progression to a neurodegenerative disorder. At the same time, altered functional connectivity from the brainstem to widespread networks, particularly those sustaining REM sleep muscle atonia, has been demonstrated (Rolinski et al., 2016; Figorilli et al., 2021; Churchill et al., 2024). Such changes are likely to affect broader systems regulating vigilance and, ultimately, circadian rhythms, given the close relationship between REM sleep and hypothalamic circadian regulation (Kunz and Bes, 1999; Kunz, 2013; Kunz et al., 2023).

Indeed, in overt α-synucleinopathies, circadian disruptions have been variably reported, from early stages throughout the disease course (Videnovic et al., 2014a; Nassan and Videnovic, 2022). Since dopamine neurotransmission is strongly implicated in circadian regulation (Korshunov et al., 2017), chronobiology has been proposed as both a relevant pathogenic domain in PD and a candidate therapeutic target (Videnovic and Willis, 2016). Blunted melatonin secretion profiles have been observed in PD and linked to clinically significant excessive daytime sleepiness (EDS) (Videnovic et al., 2014b), elevated serum cortisol, reduced melatonin, altered expression of the clock gene Bmal1, and disrupted nocturnal sleep continuity since the early disease stages (Breen et al., 2014). Moreover, across the spectrum from healthy controls to iRBD, PD, and DLB, combined actigraphy-derived rest–activity rhythms and molecular measures suggested a graded, progressive disruption of activity profiles and clock-gene expression, with melatonin appearing selectively impaired in DLB compared with both iRBD and PD (Comas et al., 2025). Furthermore, the use of exogenous melatonin as a therapeutic agent in iRBD was first proposed around the same time that iRBD was initially linked to α-synucleinopathies (Kunz and Bes, 1997), and its clinical efficacy has since been supported (McGrane et al., 2015; Kunz et al., 2021).

Hence, investigating circadian rhythm alterations in iRBD offers an opportunity to trace early changes in brain systems regulating sleep–wake behavior and internal timekeeping. Such disruptions may reflect or contribute to the neurodegenerative cascade that eventually leads to clinically overt disease, providing perspectives on both biomarkers and mechanisms of prodromal pathology and potentially informing disease-modifying strategies (Kunz et al., 2023).

In the current research landscape, two measurement approaches are currently most promising for assessing circadian functioning in prodromal α-synucleinopathies.

First, prolonged actigraphy can capture multi-night sleep–wake behavior in ecological settings, yielding both traditional sleep continuity metrics (e.g., sleep efficiency, latency, fragmentation) and circadian rest–activity indices (e.g., cosinor parameters, interdaily stability, intradaily variability, relative amplitude), allowing longitudinal tracking and large-scale deployment. Proof-of-concept studies have shown that actigraphy-derived features, such as the temporal dispersion and magnitude of nocturnal movements, can identify RBD in PD cohorts with good accuracy, supporting its feasibility as a screening and phenotyping tool in clinical contexts (Louter et al., 2014; Liguori et al., 2023; Raschellà et al., 2023). Furthermore, actigraphy has already been widely employed in aging and neurodegenerative populations to study both sleep and circadian rhythms (Videnovic et al., 2014a; Shen et al., 2023).

Second, increasingly accessible laboratory and physiological markers of circadian phase and amplitude, such as endogenous melatonin, dim-light melatonin onset (Pandi-Perumal et al., 2007), and core body temperature, provide direct readouts of central clock output and phase–behavior alignment. Although their application in α-synucleinopathies and particularly in iRBD is still at an early stage, they remain strong conceptual candidates for integration into multimodal biomarker panels (Kunz et al., 2023; Carpi et al., 2025). Moreover, neuromodulatory systems that underpin sleep–wake regulation may also intersect with circadian rhythms. For example, the orexin/hypocretin pathway, central to arousal stability, shows inconsistent stage-dependent alterations across α-synucleinopathies, with possible early compensatory increases followed by later reductions (a pattern emerging particularly in iRBD and PD), and may be better characterized with protocols that explicitly account for rest–activity phase and circadian rhythmicity (Raheel et al., 2024).

In this mini-review, the current evidence on sleep–wake rhythm alterations in prodromal α-synucleinopathies is synthesized, with a primary focus on iRBD, under the premise that network-level dysfunctions detected in iRBD can mirror those early observed in PD and DLB (Boeve et al., 2001; Boeve, 2013; Rolinski et al., 2016; McKeith et al., 2020), and emphasizing its relevance for risk stratification and for designing rhythm-targeted, disease-modifying trials. In subsequent sections, the findings from actigraphic studies of sleep and circadian indices derived from rest–activity modeling are presented first. Therefore, an overview of selected evidence from physiological assays in iRBD is provided. Conversely, consistent with the specific focus of our overview, comprehensive summaries of sleep disturbances across α-synucleinopathies which are already available in recent systematic reviews (Chahine et al., 2017; Elder et al., 2022; Pounders and McCarter, 2025) are not reiterated here.

## 2 Rest–activity rhythm alterations in iRBD and phenoconversion

Several actigraphy studies specifically investigated nighttime sleep and 24-h rest–activity rhythms in iRBD patients, but current findings are heterogeneous and not always comparable, largely due to small sample sizes and methodological differences (Liguori et al., 2023).

Regarding nighttime sleep, results are mostly inconsistent. While a well-controlled polysomnographic study comparing participants with RBD, REM sleep without muscle atonia, or dream enactment behavior to a large general population cohort did not find significant differences in sleep parameters, except for a slight reduction of deep sleep in those with probable RBD (Lee et al., 2023), some differences in nocturnal sleep emerged in prolonged actigraphy recordings. In 27 iRBD patients versus 19 elderly controls monitored for 14 days, increased time in bed and sleep latency together with reduced sleep efficiency were observed (Liguori et al., 2021). Similarly, reduced sleep efficiency and increased wake after sleep onset were reported in 19 iRBD patients compared to 16 controls assessed over 2 weeks (Filardi et al., 2020). Conversely, in a larger sample of 88 iRBD patients, 44 patients with clinically diagnosed α-synucleinopathies, and 44 controls, Feng et al. (2020) found no differences between iRBD and controls in sleep duration, efficiency, and fragmentation, and unexpectedly observed better nocturnal sleep (increased efficiency and reduced wake after sleep onset) in patients with overt α-synucleinopathies. However, the latter study did not preliminarily characterize the RBD episodes that could affect the actigraphic recordings; therefore, the reported parameters were not corrected for RBD-related activity.

Despite these inconclusive findings on conventional sleep metrics, several of the same studies also examined actigraphic measures of 24-h activity counts and diurnal sleep episodes, which more directly reflect circadian rhythmicity. In fact, actigraphy is the gold standard for tracking the sleep–wake cycle over time, providing objective, detailed information and metrics on its course and stability, rather than just monitoring conventional sleep parameters. Using cosinor and non-parametric analysis methods (Gonçalves et al., 2014), decreased circadian rhythm amplitude (Winer et al., 2023; Comas et al., 2025) and reduced diurnal activity, as reflected by both total activity and activity counts during the most active 10 h of the day (M10 index), have been consistently reported in iRBD, along with abnormally elevated nocturnal activity unrelated to RBD episodes (i.e., activity counts during the least active 5 h, L5 index) (Stefani et al., 2018; Feng et al., 2020; Liguori et al., 2021; Winer et al., 2023), likely suggesting a flattening of the day–night contrast as a characteristic trait of the disorder. Indeed, this pattern is reinforced by increased napping frequency and duration in iRBD (Feng et al., 2020; Filardi et al., 2020; Liguori et al., 2021).

Incidentally, expert visual analysis of actigraphy recordings has also been shown to reliably identify iRBD patterns, again with greater accuracy than questionnaires (Stefani et al., 2018). Moreover, a recent work has applied machine-learning models to nocturnal actigraphy data in PD cohorts and demonstrated that digital classifiers can detect RBD-related motor activity with high accuracy, supporting the potential of actigraphy as a scalable screening tool for RBD-related activity patterns (Raschellà et al., 2023).

Importantly, 24-h actigraphic measures are not only descriptive but may also carry prognostic values for disease progression from prodromal to overt stages. In Feng et al.’s (2020) cross-sectional analysis, increasing trends in napping parameters together with decreasing daytime activity distinguished controls, iRBD patients, and patients with diagnosed α-synucleinopathies. In the longitudinal analysis, iRBD patients who later progressed to PD, DLB, or MSA exhibited more pronounced daytime napping, greater daily activity fragmentation, lower M10 values, and reduced overall activity at baseline, while no differences in nocturnal sleep parameters distinguished converters from non-converters (Feng et al., 2020). Together, these findings support the notion that rest–activity dysregulation represents an early biomarker of disease progression, reflecting the progressive involvement of brainstem structures and ascending arousal pathways in the neurodegenerative process.

Nonetheless, despite these promising results, inconsistencies across studies and potential confounders must be considered when interpreting actigraphy data. Metrics such as interdaily stability and intradaily variability, which rely on prolonged monitoring, are sensitive to recording duration and analytic approach. Medications such as clonazepam and melatonin, indicated for the treatment of iRBD (Jung and St. Louis, 2016; Kunz et al., 2021; Standards of Practice Committee et al., 2010; Li et al., 2016), can significantly affect motor activity and circadian rhythms. In particular, the effects of clonazepam should be taken into account given its long half-life and its potential impact on the sleep–wake cycle via increased daytime drowsiness. Furthermore, co-occurring conditions (e.g., non-severe sleep apnea or depression), could also have an impact on actigraphy results. For instance, patients with higher depression scores showed more disrupted rest–activity cycles, suggesting that mood-related circadian effects could confound sleep–wake measures (Liguori et al., 2021). Finally, device differences and analytic algorithms further limit comparability, with some discrepancies attributable to sampling frequency or scoring rules. Moreover, actigraphy cannot directly distinguish RBD episodes from awakenings, leading to potential misclassification of nocturnal activity. Overall, these challenges underscore the need for methodological guidelines and standardized measurement pipelines in actigraphy-based research (Slyepchenko et al., 2023).

Moreover, despite growing evidence, the specific neural substrates linking altered rest–activity rhythms with prodromal neurodegeneration remain only partially characterized. Functional and neuroimaging studies suggest early involvement of brainstem and hypothalamic circuits regulating vigilance and circadian timing in iRBD (Iranzo et al., 2013; Figorilli et al., 2021; Kunz et al., 2023; Churchill et al., 2024), but causal mechanisms and directional relationships remain unclear and require further validation in prospective multimodal studies integrating actigraphic data.

In any case, actigraphic monitoring supports the view that iRBD is characterized by distinctive alterations in diurnal rest patterns and circadian rhythmicity. These changes are consistent with early neurodegenerative involvement of sleep–wake circuits, differentiate iRBD from healthy aging, and partially overlap with patterns observed in overt synucleinopathies. Together, they point to the potential of rest–activity actigraphy as a candidate biomarker for both the diagnosis of early synucleinopathies and the prediction of phenoconversion.

## 3 Laboratory-based and physiological circadian markers in iRBD

Recent studies began to characterize circadian rhythm biomarkers assessed through laboratory assays and physiological measures in iRBD, providing more direct insight into prodromal changes of the internal clock system in α-synucleinopathies. Among these, alterations in melatonin secretion rhythms have been investigated in both iRBD and established α-synucleinopathies, with promising though preliminary results.

Melatonin, a pineal hormone controlled by the hypothalamic SCN (Moore, 2007), normally exhibits a robust nocturnal rise, typically assessed through dim-light melatonin onset (DLMO; Pandi-Perumal et al., 2007). The mixed evidence on melatonin abnormalities in both PD and DLB has motivated the direct exploration of 24-h melatonin dynamics in iRBD, both in laboratory and ecological settings. For example, Weissová et al. (2018) examined blood melatonin profiles alongside clock gene expression in a pilot sample of 10 iRBD patients and nine matched controls during a 24-h laboratory protocol with three-hour interval blood sampling. Although overall melatonin rhythm and amplitude did not differ from controls, the phase of melatonin release was delayed by about 2 h in iRBD. Moreover, melatonin acrophase (peak timing) was markedly more variable across iRBD individuals (spanning an ~11-h range) compared to the tightly clustered ~five-hour range in controls, suggesting that circadian timing in iRBD is not only delayed on average but also less stable between individuals. In parallel, Weissová et al. (2018) observed molecular disruptions: peripheral blood mononuclear cells from iRBD patients lost normal circadian expression rhythms of Bmal1, Per2, and Nr1d1, and showed reduced Per3 amplitude, even though Per1 rhythmicity persisted.

More recently, Comas et al. (2025) assessed salivary melatonin and oral mucosa Bmal1 expression across four groups: DLB (*n* = 17), PD (*n* = 16), iRBD (*n* = 20), and controls (*n* = 15), using a 24-h, three-hour interval sampling protocol. While all groups retained a circadian trend for Bmal1 expression, median amplitude decreased progressively across groups (controls > iRBD > PD > DLB). For melatonin, no significant differences emerged between controls, iRBD, and PD, although a reduced amplitude and delayed acrophase trend was apparent in iRBD. In contrast, melatonin output was significantly blunted in DLB, with no evidence of rhythm oscillations across the 24-h interval.

Finally, a small real-world study tested salivary melatonin secretion collected at home across five evening hours. iRBD patients (*n* = 18) showed significantly reduced total melatonin secretion (area under the curve) compared to age-matched controls (*n* = 10) (Carpi et al., 2024). In this sample, DLMO was later on average in iRBD, although the phase delay did not reach significance, likely due to variability and the not regular sampling interval. This study also combined melatonin assays with seven-day actigraphy, revealing parallel evidence of circadian dysregulation: iRBD patients had reduced daytime activity (M10 index) and lower relative amplitude of the rest–activity cycle. Together, these findings suggest that iRBD is associated with delayed or blunted melatonin rhythms, even before overt neurodegeneration occurs during the clinical progression of the disease.

In this context, comparative findings in established α-synucleinopathies enrich the significance of these observations. As mentioned, the attenuation of melatonin amplitude in iRBD mirrors results in overt PD, where the most pronounced reductions are seen in patients with EDS (Videnovic et al., 2014b). However, phase shifts of melatonin onset have not been consistently observed in PD under controlled conditions (Videnovic et al., 2014b; Comas et al., 2025), underscoring the need for further multi-group comparisons that include prodromal iRBD alongside PD and DLB. In DLB, which often evolves from long-standing RBD, melatonin profiles remain poorly characterized despite the frequent clinical observation of severe circadian disruption (e.g., day–night sleep inversion, sundowning; Canevelli et al., 2016; Toccaceli Blasi et al., 2023), and the heavy pathology likely involving the SCN (Videnovic and Willis, 2016) makes an impairment of melatonin secretion highly expected.

Beyond melatonin, core body temperature (CBT) rhythms represent another potential circadian marker. Continuous CBT recording captures SCN-driven thermoregulatory cycles, normally peaking in the afternoon and reaching their nadir late at night. In fact, some compelling data on CBT have been reported in both overt PD and iRBD. Specifically, Zhong et al. (2013) observed a reduced CBT mesor in PD patients (*n* = 12) compared to controls (*n* = 11), along with attenuation of the nocturnal temperature decline. These CBT alterations correlated with the severity of RBD symptoms and reduced REM sleep, suggesting RBD-related brainstem pathology in thermoregulatory disruption. In a subsequent study, Raupach et al. (2020) expanded this work to patients with iRBD (*n* = 15), PD (*n* = 31), DLB (*n* = 6), and controls (*n* = 10). While no group differences in cosinor metrics emerged, nocturnal CBT amplitude was significantly reduced in iRBD, DLB, and PD presenting with RBD compared to controls, but not in PD not affected by RBD; CBT amplitude also correlated negatively with RBD symptom severity across the sample. These findings suggest that CBT changes may reflect the specific neurodegeneration underlying RBD, likely involving subcortical nuclei mediating REM sleep muscle atonia and interacting with the SCN, encouraging expanded physiological monitoring in iRBD with comprehensive circadian assay panels for clarifying these links.

Overall, while these laboratory markers are promising, methodological limitations should be considered. Existing studies typically include small samples (often 10–20 patients), raising the possibility that inter-individual variability heavily influences results. Weissová et al. (2018) reported widely dispersed melatonin peaks, with some iRBD patients showing near-normal timing and others extreme delays. Such heterogeneity may reflect different prodromal stages or genetic backgrounds, but small samples prevent firm conclusions. Protocol differences also complicate interpretation. Laboratory-based melatonin studies under constant dim light may not align with real-world salivary sampling, where ordinary light exposure could shift phase. Sampling frequency is another factor: blood draws every 2–3 h can capture a full melatonin curve, whereas limited saliva sampling risks missing DLMO or acrophase. These methodological contrasts likely contribute to inconsistent reports of melatonin phase delay (Weissová et al., 2018; Carpi et al., 2024; Comas et al., 2025). Moreover, medication and comorbidities add further complexity. Specifically, the subtle effects of exogenous melatonin, which can be used to treat RBD, could still bias results. At the same time, several commonly prescribed medications may suppress endogenous melatonin secretion and thus confound circadian assessments, with β-blockers (Stoschitzky et al., 1999) and serotonergic antidepressants (McGlashan et al., 2018) shown to reduce nocturnal melatonin levels. These effects may go unnoticed in cross-sectional protocols and contribute to interindividual variability in melatonin profiles. Finally, nearly all studies are cross-sectional, and it remains unexplored whether circadian markers worsen with phenoconversion to PD or DLB in the same individuals.

Nonetheless, current evidence supports the notion that circadian dysregulation may both reflect and contribute to neurodegenerative processes, potentially forming a recursive, bidirectional loop as illustrated in Figure 1, wherein early clock disruptions exacerbate vulnerability in neural systems involved in REM sleep regulation, thermoregulation, and arousal stability (Nassan and Videnovic, 2022; Namgyal and Lim, 2025).

In summary, laboratory measures of circadian function, ranging from melatonin secretion to CBT rhythms, provide valuable understanding of prodromal α-synucleinopathy. Current preliminary evidence suggests that clock-related dysfunction is already underway in iRBD, underscoring the possibility that circadian mechanisms contribute to the transition from RBD to overt neurodegenerative disease. However, future longitudinal, multi-modal studies are required to validate these early signals and clarify their prognostic and mechanistic significance.

## 4 Conclusion

**Figure 2:**
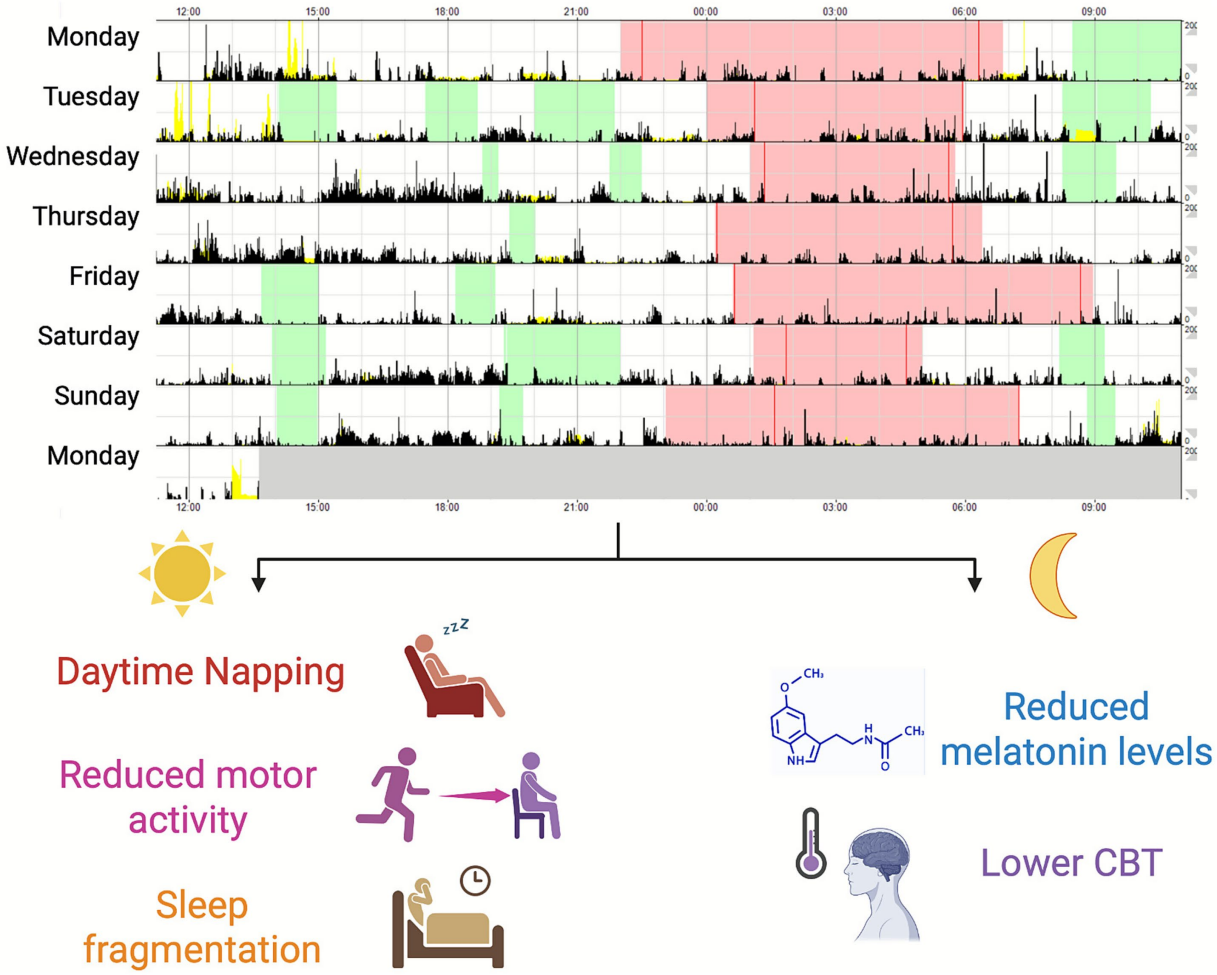
The upper part of the figure shows the traces from a week-long actigraphic recording of a representative patient diagnosed with iRBD. For each 24-h period, black bins indicate activity counts (with higher elevations reflecting greater movement), red-shaded areas denote time intervals scored as nocturnal sleep, and green-shaded areas represent daytime naps. Characteristic disruptions of sleep–wake rhythms are evident and summarized in the lower part of the figure, including frequent daytime napping, reduced daytime motor activity, and fragmented nocturnal sleep with recurrent motor activation. These alterations can be accompanied by candidate physiological biomarkers, such as reduced melatonin secretion and lower amplitude of the core body temperature (CBT) circadian rhythm. CBT, core body temperature; iRBD, isolated rapid eye movement (REM) sleep behavior disorder.

In conclusion, growing evidence highlights that, despite a largely preserved nocturnal sleep structure, patients with iRBD display circadian rhythm alterations that likely reflect early neurodegenerative changes. These alterations, including long daytime napping, disrupted rest–activity rhythms, and reduced melatonin secretion (Figure 2), not only distinguish iRBD from normal aging controls but also mirror patterns observed in manifest α-synucleinopathies, showing potential for predicting clinical phenoconversion (Feng et al., 2020). Despite the typically small sample sizes of available studies, the confirmation of core findings across independent cohorts supports their generalizability (Weissová et al., 2018; Feng et al., 2020; Liguori et al., 2021; Winer et al., 2023; Carpi et al., 2024). In this review, we underscored the most promising circadian markers of iRBD and α-synucleinopathies (see Table 1 for a summary), while acknowledging current methodological challenges that should be addressed in future studies.

**Table 1:** Candidate circadian and rest–activity biomarkers in iRBD and α-synucleinopathies.

Domain	Marker	Findings in iRBD	Comparisons with PD/DLB
Rest–activity rhythms (actigraphy)	Relative amplitude, L5/M10	Reduced amplitude, delayed phase, lower daytime activity (M10), higher nocturnal activity (L5) (Filardi et al., 2020; Liguori et al., 2021; Feng et al., 2020; Winer et al., 2023)	More pronounced disruption in PD and DLB, with greater flattening of day–night contrast (Feng et al., 2020)
Daytime behavior	Nap duration/frequency	Increased nap frequency and duration, often independent of subjective sleepiness (Filardi et al., 2020; Liguori et al., 2021; Feng et al., 2020)	No retrieved evidence on daytime napping in PD and DLB.
Melatonin secretion	DLMO timing, amplitude, variability	Delayed or dispersed DLMO; reduced secretion in ecological settings (Weissová et al., 2018; Carpi et al., 2024; Comas et al., 2025)	Reduced amplitude in PD; pronounced blunting and loss of rhythm in DLB (Videnovic et al., 2014a,b; Comas et al., 2025)
Core body temperature	24-h amplitude	Reduced nocturnal amplitude in iRBD and PD with RBD (Raupach et al., 2020)	Attenuated amplitude in DLB and PD with RBD, not in PD without RBD (Zhong et al., 2013; Raupach et al., 2020)
Clock gene expression	Bmal1, Per2, Nr1d1	Loss of rhythmicity in iRBD; reduced amplitude across markers (Weissová et al., 2018; Comas et al., 2025)	Progressive reduction from controls → iRBD → PD → DLB (Comas et al., 2025)

DLMO, dim light melatonin onset; iRBD, isolated REM sleep behavior disorder; L5, activity level during the five consecutive hours of least activity within the 24-h period; M10, activity level during the ten consecutive hours with the highest activity within the 24-h period.

Given that up to 80–90% of iRBD patients eventually develop an overt α-synucleinopathy (Savica et al., 2018; Galbiati et al., 2019), the possibility of detecting prodromal disease through sleep and circadian markers deserves clinical attention. Indeed, robust chronobiological markers in iRBD would allow stratifying high-risk individuals, monitoring disease progression, and could even serve as targets in preventive and disease-modifying trials (Videnovic and Willis, 2016; Feng et al., 2020; Kunz et al., 2023), aligning with emerging research on interventions aimed at stabilizing circadian rhythms and the use of melatonin as a chronobiotic agent (Videnovic and Willis, 2016; Pérez-Lloret and Cardinali, 2021). If found feasible and physiologically grounded, addressing circadian disruption early in iRBD may influence disease trajectories from their earliest stages, targeting initial neurodegeneration before overt pathology has been diagnosed.

However, although findings are promising, key barriers currently limit the translation of circadian biomarkers into clinical tools. In addition to the small and heterogeneous samples characterizing existing studies, major limitations include the absence of standardized protocols and the potential confounding effects of comorbidities and medications. Furthermore, substantial inter-individual variability in iRBD severity, duration, and circadian profiles must be considered (Schenck, 2022).

To accelerate clinical translation, future research should prioritize the standardization of biomarker panels (Bruschi et al., 2025), disentangle the influence of aging, comorbid conditions, and pharmacological treatments in large-scale cohorts, and clarify how circadian alterations vary across individuals and disease trajectories. Longitudinal studies are especially needed to test whether deviations from normative circadian patterns can reliably predict phenoconversion. Such efforts are essential for advancing circadian biomarkers toward real-world clinical utility in prodromal α-synucleinopathies.
